# Transcriptome Profiling Reveals the Regulatory Mechanism Underlying Pollination Dependent and Parthenocarpic Fruit Set Mainly Mediated by Auxin and Gibberellin

**DOI:** 10.1371/journal.pone.0125355

**Published:** 2015-04-24

**Authors:** Ning Tang, Wei Deng, Guojian Hu, Nan Hu, Zhengguo Li

**Affiliations:** Genetic Engineering Research Center, School of Life Sciences, Key Laboratory of Functional Gene and New Regulation Technologies Under Chongqing Municipal Education Commission, Chongqing University, Chongqing 400030, China; South China Agricultural University, CHINA

## Abstract

**Background:**

Fruit set is a key process for crop production in tomato which occurs after successful pollination and fertilization naturally. However, parthenocarpic fruit development can be uncoupled from fertilization triggered by exogenous auxin or gibberellins (GAs). Global transcriptome knowledge during fruit initiation would help to characterize the molecular mechanisms by which these two hormones regulate pollination-dependent and -independent fruit set.

**Principal Findings:**

In this work, digital gene expression tag profiling (DGE) technology was applied to compare the transcriptomes from pollinated and 2, 4-D/GA_3_-treated ovaries. Activation of carbohydrate metabolism, cell division and expansion as well as the down-regulation of MADS-box is a comprehensive regulatory pathway during pollination-dependent and parthenocarpic fruit set. The signaling cascades of auxin and GA are significantly modulated. The feedback regulations of *Aux/IAAs* and *DELLA* genes which functioned to fine-tune auxin and GA response respectively play fundamental roles in triggering fruit initiation. In addition, auxin regulates GA synthesis via up-regulation of *GA20ox1* and down-regulation of *KNOX*. Accordingly, the effect of auxin on fruit set is mediated by GA via *ARF2* and *IAA9* down-regulation, suggesting that both pollination-dependent and parthenocarpic fruit set depend on the crosstalk between auxin and GA.

**Significance:**

This study characterizes the transcriptomic features of ovary development and more importantly unravels the integral roles of auxin and GA on pollination-dependent and parthenocarpic fruit set.

## Introduction

Fruit set, defined as the shift from the quiescent ovary to fast-growing young fruit, is a key process for fruit production in flowering plants. Owing to its economic importance as a fleshy crop, tomato (*Solanum lycopersicum* L.) has been widely studied as a model for the regulation of these processes. Generally, fruit set occurs after the successful completion of pollination and fertilization, while it is coordinated by signals produced in the developing embryos [1]. Auxin and gibberellins (GAs) are considered the major phytohormones in the control of this process [2], which is supported by the elevated levels of endogenous auxin and GA in pollinated ovaries [1, 3] and parthenocarpy induction via exogenous application of either of the two hormones uncoupling with pollination and fertilization [4], even though others have been shown to be involved in fruit set such as ethylene [5] and cytokinin [6].

Over the years, it has been well established that the fruit developmental program is triggered by the signaling cascades of auxin and GAs. Recent studies have identified several components of the auxin signaling pathway including auxin transport protein PIN4[7], the Aux /IAA protein IAA9 [8], auxin response factor ARF8[9] and ARF7[10] involved in repressing fruit initiation until the fertilization cue in tomatoes. In addition to auxin signaling, fruit set seems to be under the control of GAs biosynthesis and signaling. In pollinated ovaries, the increase of GA content is attributed to higher GA 20-oxidase (GA20ox) activity via elevated GA20ox transcripts [3], hence promotes GA response. DELLA protein has been characterized as a negative regulator of GA signal and thereby prevents ovary growth prior to pollination and fertilization [2]. Although it is suggested that fruit set depends on auxin and GA response by the above-mentioned results, the exact roles of these two hormones are still partially defined, probably due to only a few signaling components involved have been identified to date [2]. Additionally, despite the application of either auxin or GAs can trigger parthenocarpy in tomato, there are several indications that each of them has distinct effects on fruit development such as cell division and cell expansion [1, 11]. Therefore, global transcriptome knowledge during fruit set might help to understand the molecular mechanisms by which these two hormones regulate fruit set and development.

More recently, some advances have brought some insights on their regulatory roles. Vriezen et al. [5] compared the transcriptomes from pollinated and GA-treated ovaries 3 days after treatment. It showed that genes triggered by one treatment were not all triggered by another, which is supported by the fact that pollination appeared to have significant effects on the expression of the auxin signaling genes, such as *Aux/IAAs*, while these genes were hardly influenced by GA treatment. By contrast, a transcriptome analysis underlying pollination-dependent fruit set and parthenocarpy mediated by *IAA9* down-regulation revealed that a large number of genes were differentially expressed in common, and only a small subset dependent on *IAA9* regulation. This study also showed that some molecular events including the activations of photosynthesis, sucrose metabolism and cell division were considered to be important in both types of fruit set during anthesis to postanthesis transition [12]. Similar results are also revealed by transcriptomic analysis of carpel development in *pat3/ pat4* mutant with higher GA concentrations [4]. These findings suggest that regardless of the existence of distinctive molecular features, different types of fruit set could be regulated via a similar pathway in partially. Furthermore, the roles of auxin and GAs on fruit set are demonstrated in these studies. Wang et al. [12] shows that the fine-tuning of auxin-related *Aux/IAAs* and *ARFs* are crucial in triggering fruit set, whereas few changes concerning GA appear comparatively. Similarly, auxin biosynthesis and responses were altered in *pat3/ pat4* mutant [4], indicating the complexity of auxin action in fruit set. Alternatively, it has been reported that auxin-induced parthenocarpic fruit set in tomato is mediated via GAs in partially, which is indicated by the observation that fruit set induced by 2, 4-D was dramatically repressed by GA biosynthesis inhibitors [13]. The above-mentioned studies demonstrate the complicated and confusing relationship between auxin and GA response during fruit set. It is helpful to clear the crosstalk between these two hormones and the comprehensive mechanisms of fruit set.

In order to characterize the molecular events underlying the fruit set, digital gene expression tag profiling (DGE) technology was applied to compare the transcriptomes from pollinated, 2, 4-D and GA_3_-treated ovaries in this work. Our study identified the genes modulated throughout carpel development and revealed common and distinct features between pollination-dependent and parthenocarpic fruit set. The signaling cascades of auxin and GAs and potential components of the regulatory mechanism underlying different types of fruit set processes were discussed. A model integrating the role of auxin and gibberellin in tomato fruit set was established in our study.

## Results

### Transcriptomic analysis of ovary development during fruit set

**Fig 1:**
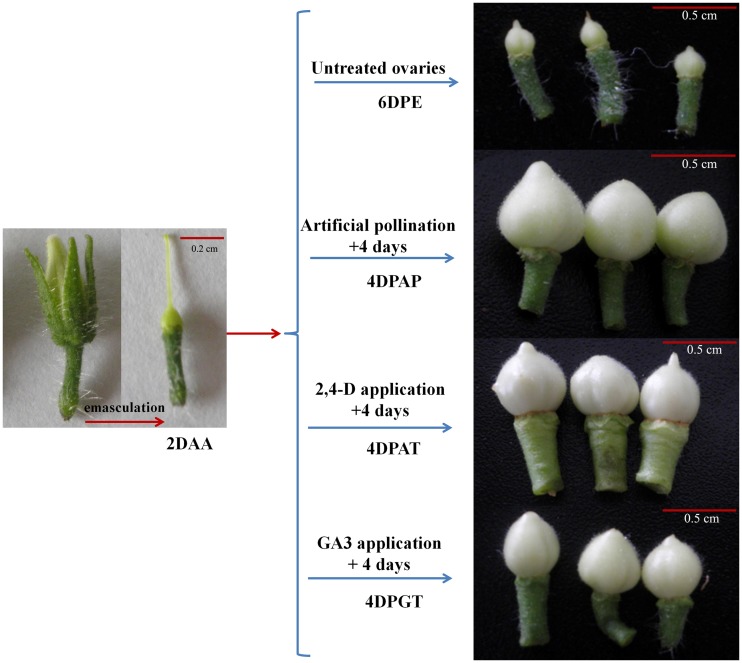
Ovaries collection for analyzing transcriptomic changes underlying fruit set in tomato. Flowers were emasculated at 2 days before anthesis (2DAA), followed by different treatments on the day just equivalent to anthesis. Then, the uniform ovaries were collected 4 days after pollination (4DPAP), 2,4-dichlorophenoxyaceticacid (2,4-D) (4DPAT) and gibberellin application (4DPGT), while the untreated ovaries were considered as control (6DPE).

To characterize transcriptomic profiles of ovary development during pollination-dependent and-independent fruit set, five libraries including ovaries at 2 days ahead of anthesis (2DAA), 4 days post artificial pollination (4DPAP), auxin treatment (4DPAT), GA_3_ treatment (4DPGT) and untreated ovaries (6DPE) were designed for RNA-Seq analysis (Fig 1). The raw data generated by sequencing for the libraries ranged from 3.49 to 3.68 million reads. After filtering, we obtained an average of 3.45 million clean data and the number of distinct tags is from 83,342 to 116,726 (Table 1). The results of saturation evaluation and the distribution analysis of RNA-Seq tags showed that sequencing quality was high enough for gene expression analysis (S1 Fig). A SGN unigene database containing 42257 unigenes was used to tag alignment. Statistics analysis showed that the number of unambiguous tags mapping to gene was between 45620 and 62480, accounted for 53.32% to 55.17% of the clean tags (Table 1). In total, 18705 genes were detected in at least one of the five libraries, of which 11075 genes were expressed in all the samples. Moreover, 15111, 13921, 13522, 13499 and 16446 genes were expressed in ovaries at 2DAA, 4DPAP, 4DPAT, 4DPGT and 6DPE, respectively.

**Table 1:** Summary of the RNA-Seq data in ovaries during fruit set.

Summary		2DAA	4DPAP	4DPAT	4DPGT	6DPE
Raw Data	Total	3511331	3582739	3680921	3493203	3560630
Raw Data	Distinct Tag	208288	187850	179986	180556	243899
Clean Tag	Total number	3398631	3478442	3580876	3393539	3431151
Clean Tag	Distinct Tag number	98735	86704	83342	84004	116726
Unambiguous Tag Mapping to Gene	Distinct Tag number	53394	46234	45978	45620	62480
Unambiguous Tag Mapping to Gene	Distinct Tag % of clean tag	54.08%	53.32%	55.17%	54.31%	53.53%
Unknown Tag	Total number	140546	139117	116197	141453	147641
Unknown Tag	Total % of clean tag	4.14%	4.00%	3.24%	4.17%	4.30%

### Differentially expressed genes during fruit set

**Fig 2:**
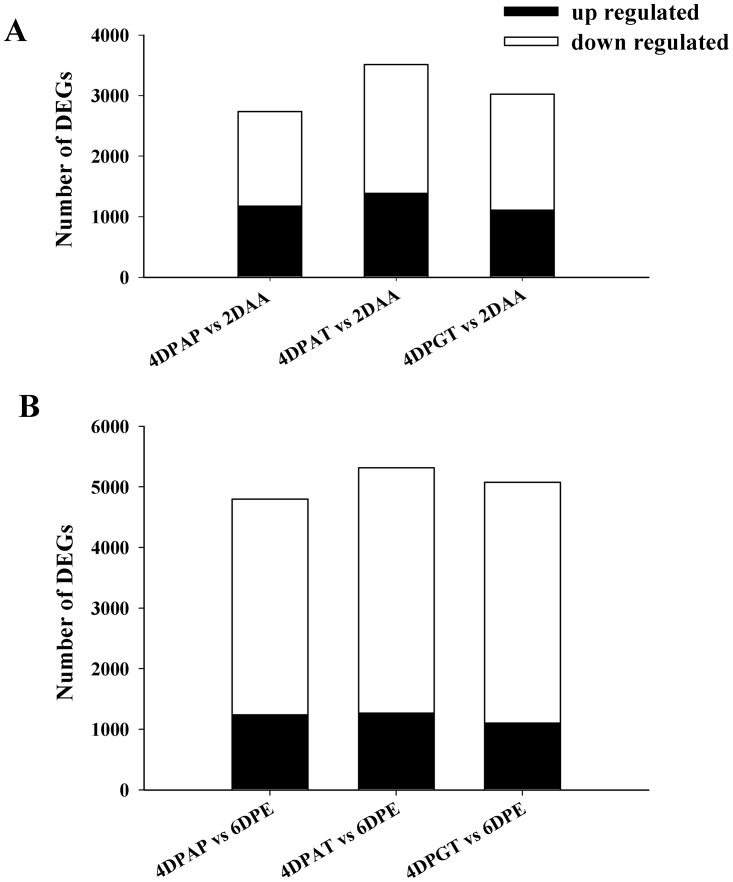
Differentially expressed genes identified by RNA-Seq during fruit set. The number of up- and down-regulated DEGs in ovaries at 4DPAP, 4DPAT and 4DPGT with respect to (A) 2DAA and (B) 6DPE, respectively during fruit set.

With respect to 2DAA, 2736, 3511 and 3025 genes were differentially expressed in ovaries at 4DPAP, 4DPAT and 4DPGT, respectively (Fig 2A, S1 Table). In addition, with respect to 6DPE, 4800, 5315 and 5077 genes were differentially expressed in ovaries at 4DPAP, 4DPAT and 4DPGT, respectively (Fig 2B, S2 Table). We also found that the majority of differentially expressed genes (DEGs) are down regulated during the process of ovary development (Fig 2).

The DEGs were assigned to one of 23 GO terms based on biological process (S2 Fig). The terms “metabolic process” (around 25%), “cellular process” (around 19%), “response to stimulus” (around 10%) and “biological regulation” (around 5.5%) stood for the major proportion. Furthermore, significantly altered KEGG pathways were identified during fruit set, of which the common enriched was plant hormone signal transduction among the six groups (data not shown).

**Fig 3:**
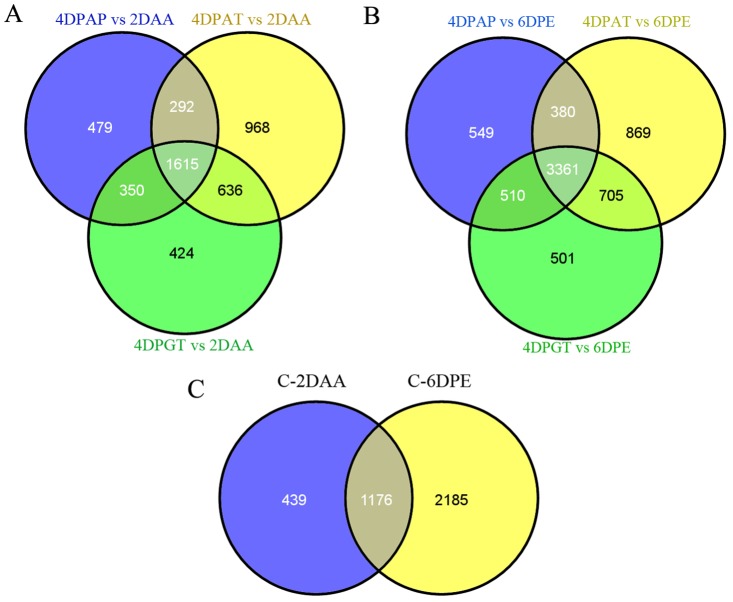
Venn diagram of DEGs associated with fruit set. The common and distinct DEGs during the three types of fruit set with respect to two controls, 2DAA (A) and 6DPE (B), respectively. (C) The DEGs common to all the three types of fruit set with respect to both controls. C-2DAA and C-6DPE represent common DEGs with respect to 2DAA and 6DPE, respectively.

To identify the common features and differences between pollination-dependent and-independent fruit set, we compared the sets of DEGs among the comparison groups based on two controls (2DAA and 6DPE). The results showed that 1615 and 3361 genes were common differentially expressed in ovaries at 4DPAP, 4DPAT and 4DPGT in contrast with 2DAA and 6DPE, respectively (Fig 3A and 3B, S3 Table).

Additionally, there were 1176 genes differentially expressed in ovaries from 3 different types of fruit set in contrast with both controls, which were considered to be key regulators for fruit set and growth (Fig 3C, S3 Table). Moreover, with respect to 2DAA, 479 genes exhibited differential expression exclusively in ovaries at 4DPAP (S3 Table), indicating a correlation between these genes and seed formation. 2028 genes were identified that are only differentially expressed in parthenocarpic fruit set, of which 968 and 424 displayed auxin-dependent and GA-dependent regulation, respectively (S3 Table). Similar results were obtained based on the control 6DPE.

**Fig 4:**
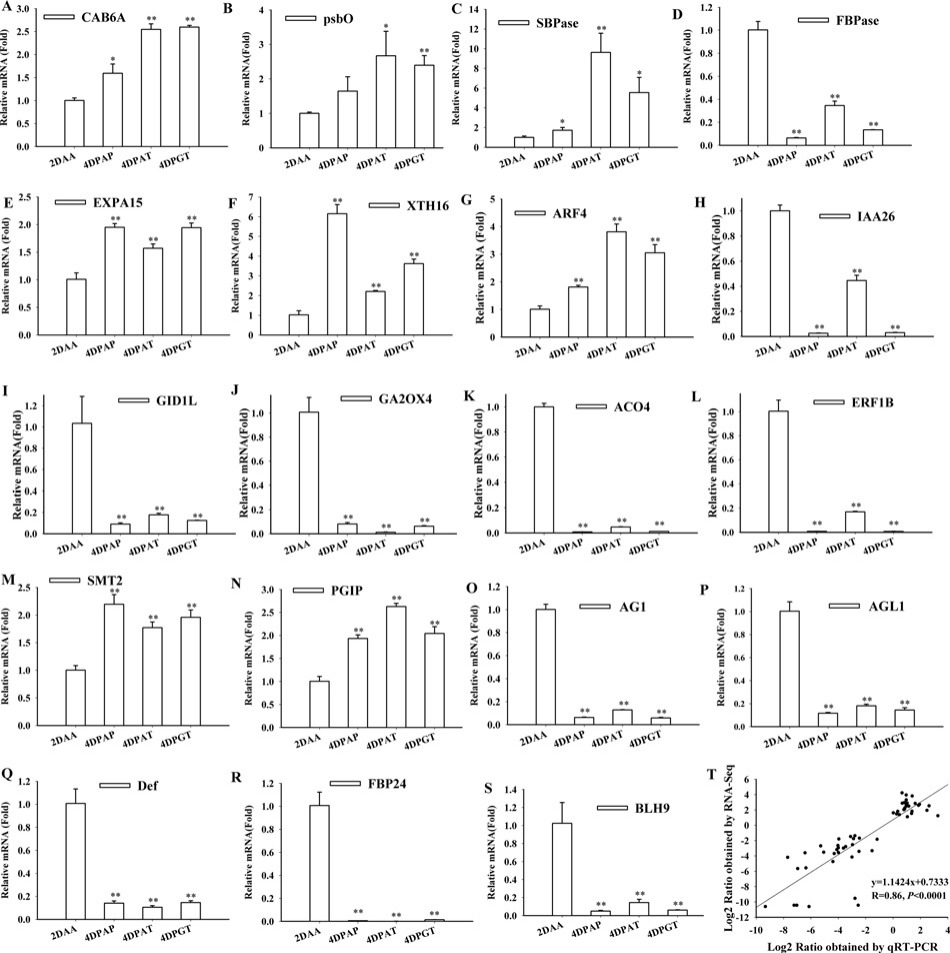
Expression patterns of selected genes identified by RNA-Seq were validated by qRT-PCR. Expressions of selected genes related to photosynthesis (A) *CAB6A* and (B) *psbO*, carbohydrate metabolism (C) *SBPase* and (D) *FBPase*, cell expansion (E) *EXPA15* and (F) *XTH16*, auxin signaling (G) *ARF4* and (H) *IAA26*, GA metabolism and signaling (I) *GID1* and (J) *GA2OX4*, ethylene biosynthesis and signaling (K) *ACO4* and (L) *ERF1B*, BR regulation(M) *SMT2* and (N) *PGIP*, MADS-Box proteins (O) *AG1*, (P) *AGL1*, (Q) *Def* and (R) *FBP24*, homeobox (S) *BLH9* in ovaries at 4DPAP, 4DPAT and 4DPGT were analyzed by qRT-PCR. Relative expression levels were determined based on the reference 2DAA set to 1. Error bars represent means ± SE (n = 3). Statistical significance was carried out by student’s t-test. Different asterisks indicate different significant levels, one asterisk (*) for *P* < 0.05 and two asterisks (**) for *P* < 0.01. (T) RNA-Seq validation by qRT-PCR. Correlation between the RNA-Seq data and the qPCR results were revealed by regression analysis.

To validate the accuracy of the RNA-Seq data, 19 genes with different functions including carbon assimilation, cell division and hormone signaling were selected for qRT-PCR analysis (Fig 4). There was a good correlation (R = 0.86, *P*<0.0001) between the two methods (Fig 4T), indicating the high reliability of the RNA-Seq data obtained in this study.

### Expression of genes involved in photosynthesis and carbohydrate metabolism during fruit set

Photosynthesis-related genes were strongly stimulated during fruit set. Remarkably, the overwhelming majority of these genes (28 out of 29 genes) were up-regulated in ovaries at 4DPAP,4DPAT and 4DPGT compared with 2DAA and 6DPE,which including 13 chlorophyll a/b-binding proteins (*CAB*), 3 photosystem I reaction center subunits (*psa*), 5 photosystem II reaction center proteins (*psb*) and 2 membrane proteins (S4 Table). The mRNA levels of *CAB6A* and oxygen-evolving enhancer protein 1 (*psbO*) were validated by qRT-PCR, which were elevated in ovaries at 4DPAP, 4DPAT and 4DPGT (Fig 4A and 4B), and additionally, their upward trends remained in 10 days after artificial pollination (S3 Fig). Furthermore, 43 DEGs associated with carbohydrate metabolism were identified. The genes encoding carbohydrate assimilation and sugar metabolism enzymes showed increased transcripts in ovaries at 4DPAP, 4DPAT and 4DPGT, e.g. sedoheptulose-1,7-bisphosphatase (*SBPase*), invertase, hexokinase-1 (*HXK1*) and fructokinase-2(*Frk2*) (Fig 4C, S4 Table). Sugars produced from assimilation could be further degraded through the glycolysis and TCA cycle to generate energy. Several genes involved in these processes were up-regulated after pollination and hormone supply, e.g. fructose-bisphosphate aldolase (*FBA*), pyruvate dehydrogenase (*PDH*), pyruvate kinase (*PK*) and ATP-citrate synthase(*ACLY*). In addition, two sugar transporter family proteins including a hexose transporter and an anion transporter 2 (*ANTR2*) displayed up-regulation during fruit set (S4 Table). The results suggested that photosynthesis and carbohydrate metabolism play important roles in onset of both pollination-dependent and parthenocarpic fruit development.

### Expression of genes associated with cell division and cell wall during fruit set

After pollination or hormone application, cell division and cell wall related genes in ovaries were strongly induced (S5 Table). Of cell division related DEGs, 12 (one G2/mitotic-specific cyclin (*CYCB1; 2*), two cyclin dependent kinases and nine histones) were up-regulated in ovaries at 4DPAP, 4DPAT and 4DPGT. In addition, fruit growth was accompanied by cell wall biosynthesis and expansion. S5 Table listed 21 cell wall associated DEGs during fruit set, which including expansin, xyloglucan glycosyltransferase, pectinesterase and glucanase, among them vast majority (17/21) were up-regulated. Detailed evaluation by qRT-PCR displayed the elevated expression of *EXPA15* and *XTH16* in ovaries at 4DPAP, 4DPAT and 4DPGT (Fig 4E and 4F), coinciding with the RNA-Seq results. It’s obvious that the changes in cell division and cell wall metabolism are fundamental events during fruit growth.

### Hormone metabolism and signaling during fruit set

**Fig 5:**
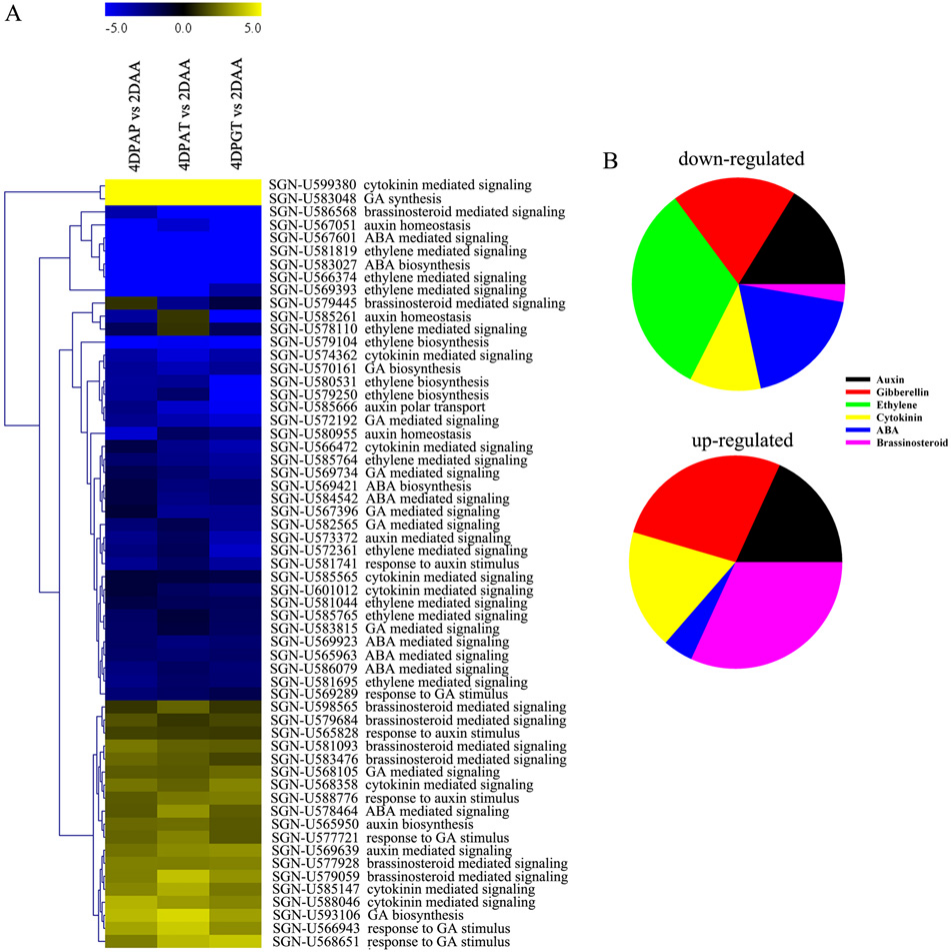
DEGs associated with hormone biosynthesis and signaling. (A) Cluster analysis of DEGs associated with hormone biosynthesis and signaling. (B) Pie charts show the percentages of up- and down-regulated DEGs involved in hormone biosynthesis and signaling during fruit set.

Up to 59 and 84 DEGs related to hormones were screened during fruit set with respect to 2DAA and 6DPE, respectively (Fig 5A, S4 Fig and S6 Table). These genes were involved in the biosynthesis and signaling of auxin, GA, ethylene, ABA, brassinosteroid and cytokinin, among which the former three were the most prominent (Fig 5B), indicating the important roles in coordinating fruit set.

As well-known regulator in fruit set, 10 auxin related genes displayed dramatic changes in their expressions during all the three types of fruit set with respect to 2DAA (S6 Table). Among them, three *IAA-amido synthases* (*GH3*.*1*, *GH3*.*6*), *SAUR-AC1*, *IAA26*, and *AUX1* were down-regulated, while anthranilate synthase beta subunit (*ASB1*), *ARG7* and *ARF4* genes were up-regulated in ovaries at 4DPAP, 4DPAT and 4DPGT (Figs 5A, 4G and 4H, S6 Table). In particular, the expression of *ARF4* was increased gradually until at least 10 days post artificial pollination, while *IAA26* decreased sharply from the anthesis stage to 6 DPAP, following a slight ascent (S3 Fig). Of the 59 modulated genes related to hormones, 13 were assigned to GA regulation (Fig 5A). Both pollination and hormones application increased the expression of a GA biosynthesis gene, *GA20ox1* and a GA receptor gene, *GID1L2* (SGN-U568105). Remarkably, auxin treatment induced the strongest up-regulation of *GA20ox1*, which exhibiting 10–20 folds higher level of expression than that by pollination or GA_3_ treatment. Whereas, a GA 2-oxidase gene (*GA2ox4*), associated with GA catabolism, was down-regulated dramatically during fruit set, which continued until at least 10 days post artificial pollination (Fig 4J, S3 Fig). Furthermore, some GA responsive proteins were down-regulated e.g. *GRAS9* and *GRAS6*, while others displayed up-regulation e.g.*GASA1*, *RSI-1* and *GAST1* during fruit set (S6 Table). Ethylene was known to have potential role during fruit set. RNA-Seq data showed a decrease in the mRNA levels of all ethylene related genes (Fig 5A and 5B). Three ethylene biosynthesis genes (ACC oxidase, *ACO*, *ACO2* and *ACO4*) and 9 ethylene signaling genes including *CTR*, *ETR*, *EIN3*, *EBF* and *ERFs* appeared down-regulation in ovaries at 4DPAP, 4DPAT and 4DPGT (Fig 5A, S6 Table). Validation by qRT-PCR showed that the mRNAs of *ACO4* and *ERF1B* stayed a very low level after fruit initiation (Fig 4K and 4L), suggesting a relationship between the decrease of ethylene response and fruit set.

### Identification of transcription factors associated with fruit set

**Fig 6:**
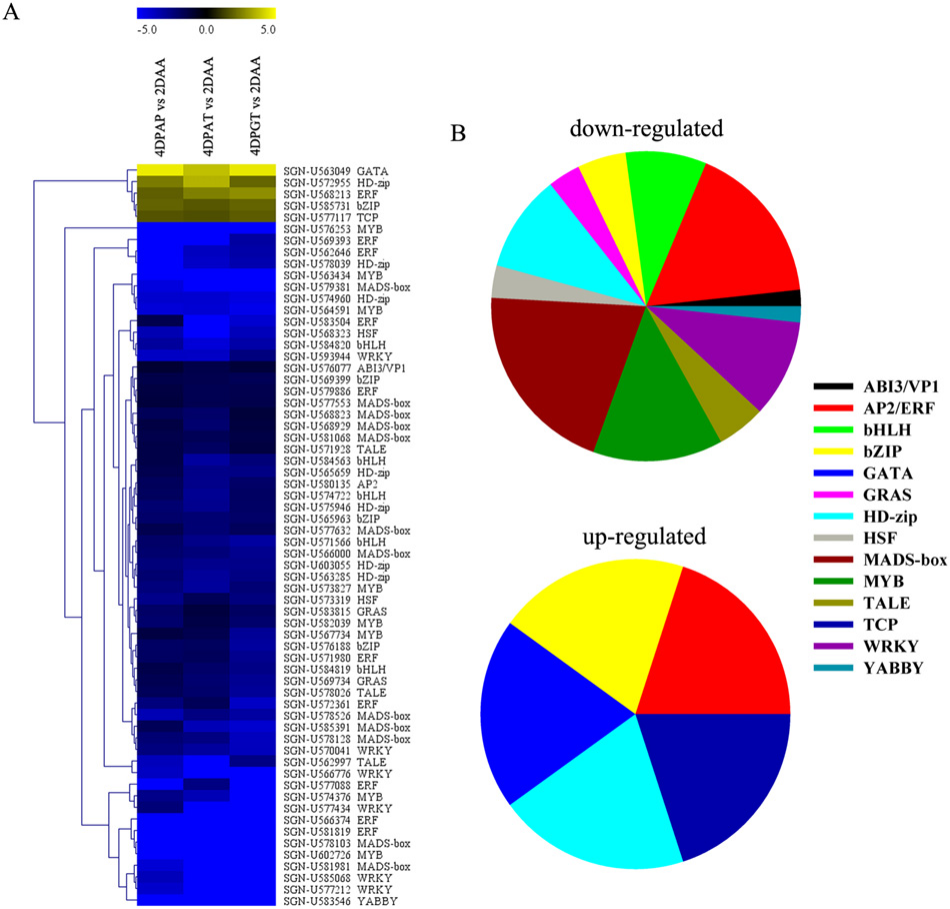
DEGs associated with transcription factors. (A) Cluster analysis of DEGs associated with transcription factors. (B) Pie charts show the up- and down-regulated TFs during fruit set.

RNA-seq data showed that 64 and 99 transcription factors displayed differential expression with respect to 2DAA and 6DPE respectively, and the vast majority of them were down-regulated during fruit set (Fig 6A, S5 Fig). These TFs were classified into several families including MADS-box, HB (HD-Zip and TALE), AP2/ERF, bZIP, MYB, bHLH, WRKY and GRAS, among which MADS-box, HB and AP2/ERF family recruited the major members (Fig 6B). 13 MADS-box genes displayed down-regulation in ovaries at 4DPAP, 4DPAT and 4DPGT during fruit set (Table 2). QRT-PCR analysis showed that the transcripts of four MADS-box genes, *Agamous1* (*AG1*), *Agamous-like 1* (*AGL1*), *Deficiens* (*Def*) and *FBP24*, were in dramatic declines after pollination or auxin/GA_3_ treatments (Fig 4O–4R). Meanwhile, there were 9 HB superfamily members (6 for HD-Zip and 3 for TALE) differentially expressed and the overwhelming majority (8/9) was down-regulated. Further evaluated by qRT-PCR showed that a TALE family member, BEL1-like homeodomain protein 9 (*BLH9*), underwent a down-regulation during fruit set (Fig 4S). It can be noted that these TFs, especially MADS-box and HB family may play key roles in both pollination-dependent and-independent fruit set and function via down-regulating their transcription.

**Table 2:** DEGs involved in MADS-box and homeobox family during fruit set.

SGN accession number	TF family	4DPAP/2DAA	4DPAT/2DAA	4DPGT/2DAA	Gene annotation
SGN-U578526	MADS-box	-3.55	-2.54	-3.17	AG1
SGN-U568929	MADS-box	-1.32	-2.16	-1.25	TDR6
SGN-U581481	MADS-box	-2.04	-3.14	-2.00	TM29
SGN-U568823	MADS-box	-1.73	-2.08	-1.14	Def
SGN-U578103	MADS-box	-9.13	-9.13	-9.13	JOINTLESS
SGN-U577632	MADS-box	-1.56	-2.28	-1.82	MADS11
SGN-U579381	MADS-box	-4.37	-5.14	-5.36	MADS-box protein 6
SGN-U577553	MADS-box	-1.21	-1.59	-1.47	SEPALLATA3
SGN-U578128	MADS-box	-2.29	-2.54	-3.73	fruitfull1
SGN-U581068	MADS-box	-1.48	-1.69	-1.36	AGL1
SGN-U585391	MADS-box	-1.74	-3.57	-4.05	AGL11
SGN-U566000	MADS-box	-2.16	-2.37	-2.79	MADS3
SGN-U581981	MADS-box	-4.18	-10.6	-10.6	MADS29
SGN-U562997	TALE	-3.69	-9.52	-2.64	BLH9
SGN-U571928	TALE	-1.42	-1.91	-1.29	LeT12
SGN-U578026	TALE	-1.76	-2.09	-2.99	LeT6
SGN-U563285	HD-zip	-2.2	-3.03	-2.58	HB-40
SGN-U565659	HD-zip	-1.57	-2.69	-2.61	HDG2
SGN-U578039	HD-zip	-10.3	-3.91	-3.42	HAT5
SGN-U574960	HD-zip	-4.06	-4.09	-4.44	HB-13
SGN-U572955	HD-zip	2.439	3.548	2.001	HB-15
SGN-U575946	HD-zip	-2.21	-2.8	-1.97	GLABRA2

The fold-change value is represented by the Log2 Ratio.

### Strongly regulated auxin and GA signaling genes revealed differences between pollination-dependent and parthenocarpic fruit set

Considering the prominent role of auxin in the process of fruit set, we sought to identify the expression profiles of *Aux/IAAs* and *ARFs* in 4-day-old ovaries after pollination and 2,4-D /GA_3_ treatments with respect to 2DAA (Table 3). Throughout pollinated-dependent fruit set, some *ARFs* and *Aux/IAAs* displayed dramatic shifts in their expressions. These genes including *ARF4*, *ARF9-1*, *ARF9-2*, *IAA2*, *IAA12*, *IAA13* and *IAA14* were up-regulated and the enhanced expressions of *Aux/IAAs* could be needed to create a negative feedback loop[2]. Notably, 2, 4-D induction resulted in a similar regulation of these *ARFs* and *Aux/IAAs*. Others including *ARF2*, *ARF5* and *IAA26* underwent down-regulation in pollinated and GA_3_-treated ovaries. Interestingly, pollination and GA_3_ treatment inhibited the mRNA levels of *IAA9*, which were more strongly suppressed with respect to 6DPE than 2DAA (Table 3, S2 Table). In the case of *IAA9* transcripts were higher in ovaries at 6DPE, it showed flat in 2,4-D induced ovaries (S2 Table).

**Table 3:** Expression of genes associated with auxin and GA signaling in ovaries after pollination and 2,4-D/GA_3_ treatments.

SGN accession number	4DPAP/2DAA	4DPAT/2DAA	4DPGT/2DAA	Gene annotation
SGN-U568849	-1.50	1.21	-0.58	IAA9
SGN-U569639	2.26	2.73	2.85	ARF4
SGN-U571269	-1.90	-	-5.25	ARF5
SGN-U573372	-2.69	-1.82	-3.51	IAA26
SGN-U574088	-	-	-1.04	ARF8B
SGN-U576826	4.85	4.35	-	ARF9-1
SGN-U577682	-	1.15	-	IAA6
SGN-U579354	2.97	4.57	-	IAA13
SGN-U579607	-	1.64	-	IAA19
SGN-U579618	1.92	3.21	-	IAA14
SGN-U579795	1.49	2.78	-	IAA12
SGN-U579928	-1.33	-	-1.69	ARF2
SGN-U580599	9.30	8.61	-	ARF9-2
SGN-U586760	-	2.34	1.92	IAA36
SGN-U592947	-	9.26	-	IAA7
SGN-U599474	3.96	5.30	-	IAA2
SGN-U566795	-	-1.53	-3.24	SCL13
SGN-U568105	1.82	1.76	2.12	GID1L2
SGN-U568385	-	-	-1.6	SCL14
SGN-U569734	-1.6	-2.19	-2.85	GRAS6
SGN-U572192	-2.79	-3.42	-4.15	GID1L2
SGN-U575114	-	1.27	1.03	GAI
SGN-U575808	-	1.00	2.03	GID1L3
SGN-U580033	-	-	-1.45	GRAS7
SGN-U581650	-2	-	-2.97	GID1B-like
SGN-U581883	-	-3.77	-2.7	GID1-like
SGN-U583815	-2.01	-1.25	-1.91	GRAS9
SGN-U585152	-	-2.08	-1.19	GRAS1
SGN-U585573	-1.77	-	-1.23	GID1L3

In contrast with ovaries at 2DAA or 6DPE, DEGs associated with GA signaling were screened in ovaries 4 days after pollination or 2,4-D / GA_3_ treatment (Table 3). In particular, several genes were suppressed by GA treatment alone, such as *SCL14* and *GRAS7*. Two GA receptors, *GID1-Like* (SGN-U581650 and SGN-U585573), were significantly down-regulated during pollination and GA induced fruit set. In addition, a number of genes were modulated by both GA and auxin treatment, e.g. *SCL13* and *GRAS1*with down-regulation and *GAI* with up-regulation, indicating that these genes may be associated with parthenocarpy (Table 3).

### Differentially expressed genes associated with parthenocarpy

RNA-Seq data indicated that throughout the process of auxin induced fruit set, a subset of genes displayed dramatic changes in their expressions (Table 4). Briefly, auxin treatment resulted in a strong up-regulation of an auxin transporter *LAX2* and 3 *Aux/IAAs* (*IAA19*, *IAA6* and *IAA7*), while sharply reduced the transcripts of a MADS-box protein *AGL66* and a KNOX gene *KNAT3*. Also, DEGs related to parthenocarpy only assigned to the GA treatment were screened (Table 4). GA application decreased the expression of several genes including *GA2ox1*, *GID2* and *GRAS*. Moreover, it’s interesting that the transcription level of *ARF8B* was inhibited by GA application alone, which showed no changes in ovaries at 4DPAP and 4DPAT with respect to 2DAA (S1 Table). A functionally unknown *Aux/IAA* gene, *IAA36*, was identified to be up-regulated in both auxin and GA induced ovaries. Furthermore, two argonaute genes (*AGO5* and *AGO10*) and a transcription factor *BLH1* were significantly down-regulated, implying their potential involvement in the process of parthenocarpic fruit set (Table 4).

**Table 4:** DEGs associated with parthenocarpy induced by auxin and GA.

SGN accession number	4DPAT/2DAA	4DPGT/2DAA	Gene annotation
SGN-U579607	1.64	-	AUX/IAA family protein,IAA19
SGN-U577682	1.15	-	AUX/IAA family protein,IAA6
SGN-U592947	9.26	-	AUX/IAA family protein,IAA7
SGN-U583242	8.61	-	Auxin transporter-like protein, LAX2
SGN-U569018	9.39	-	SAUR, auxin-induced protein 6B
SGN-U573534	4.91	-	Probable IAA-amido synthetase GH3.1
SGN-U585261	1.1	-	Probable IAA-amido synthetase GH3.1
SGN-U572630	2.12	-	homeobox protein knotted-1 like 1 (KN1)
SGN-U571929	-2.2	-	homeobox protein knotted-1 like 3 (KNAT3)
SGN-U569575	-2.1	-	MADS-box protein AGL66
SGN-U574088	-	-1.04	auxin-responsive factor (ARF8B)
SGN-U572513	-	-1.2	SLEEPY1 protein,GID2
SGN-U597732	-	-1.38	gibberellin 2-oxidase,GA2ox1
SGN-U572163	-	-1.53	Gibberellin-regulated protein 6
SGN-U580033	-	-1.45	GRAS family, GRAS7
SGN-U568385	-	-1.56	GRAS family, SCL14
SGN-U604674	-	-8.78	homeobox family protein,HDG5
SGN-U563343	-8.78	-8.8	Argonaute protein,AGO10
SGN-U562884	-1.09	-1.2	Argonaute protein,AGO5
SGN-U586760	2.34	1.92	AUX/IAA family protein,IAA36
SGN-U578015	-2.74	-1.3	BEL1-like homeodomain protein 1,BLH1
SGN-U575114	1.27	1.03	DELLA protein, GAI
SGN-U581883	-3.77	-2.7	gibberellin receptor,GID1-like
SGN-U575808	1	2.03	gibberellin receptor,GID1L3
SGN-U585152	-2.08	-1.2	GRAS family,GRAS1
SGN-U566795	-1.53	-3.2	GRAS family, SCL13

## Discussion

Recently, several studies have been focused on fruit set, however, the regulatory mechanisms associated with pollinated-dependent and-independent fruit set are still plausible [4, 5, 12]. Our aim is to identify transcriptomic changes occurring during the ovary development after fertilization and hormone application including auxin and GA_3_, in order to perceive the common features and differences between pollination-dependent and parthenocarpic fruit set.

### The common molecular features between pollination-dependent and parthenocarpic fruit set

Global transcriptome profiling identified a great many genes showed differential expression in the developing ovaries under different treatments. Among them, approximately half were common to both fertilization-dependent and parthenocarpic fruit set, which revealing that some conserve mechanisms occur in varies fruit set processes.

Photosynthesis and carbohydrate metabolism related genes showed strongly accumulation during both pollinated-dependent and-independent fruit set (S4 Table). Although recent study holds the opinion that metabolites for fruit growth are predominantly imported from source tissues [14], the green fruit pericarp of tomato is photosynthetically active[15]. Kolotilin et al [16] showed that the induction of genes associated with photosynthesis positively correlate with cell size in tomato pericarp cells. Our result shares the viewpoint that fruit photosynthesis plays an important role in fruit establishment [12]. Plant reproduction depends largely on a sufficient import of photoassimilates, mainly sucrose [17]. In tomato plants, the major sugars including sucrose, glucose and fructose were found accumulated in ovaries after fertilization [12]. Previous reports showed that higher levels of sucrose and starch accumulated via overexpression of *SBPase* in plants[18]. Accordingly, up-regulation of *SBPase* displayed after pollination and hormone application in our study (Fig 4C). Indeed, maintaining the sucrose supply and utilization, that is, degradation into hexoses is a key to sustain the fruit set developmental process. The role of invertase in regulating sucrose metabolism has long been postulated[19]. RNAi-mediated silencing of an invertase gene, *Lin5*, aggravated fruit abortion in tomato [20], and vice versa [21]. Here, we reported that an *invertase* gene (SGN-U579335) was up-regulated in ovaries after pollination and hormone treatments (S4 Table). The results seemingly confirm the theory that glucose generated by invertase serves as a signal to repress programmed cell death (PCD) and to promote cell division which lead to fruit set [17]. Fructokinase (Frk), as a limited enzyme in sucrose metabolism, was significantly up-regulated during fruit set (S4 Table), which is in accordance with a recent study that *Frk* gene suppression resulted a reduction in flower development and fruit setting in tomato[22], implying its significant role in fruit development. Moreover, sugar metabolism may be regulated by the up-regulation of *ARF4* (Fig 4G) during fruit set [23].

Down-regulation of a large number of transcription factors is another striking feature during fruit set (Fig 6A, S5 Fig). Among them, MADS-box and homeobox genes were the most represented members, especially the former (Table 2). Notably, several *MADS-box* genes identified by RNA-Seq have been functionally characterized. Mazzucato et al.[24] indicated a role for *SlDef* in the control of fruit set. Down-regulation of *AG1* displays the replacement of carpels with pseudocarpels bearing indeterminate floral meristems and strong reduction of seed production [25]. Similar result occurs in *FBP24* that the knockdown line produces a few seeds [26]. In addition, down-regulation of *TM29* produces infertile pistil coupled with parthenocarpic fruit formation[27]. In total, we agree with the hypothesis that the low expression of MADS-box promotes fruit set even in the absence of fertilization[12]. However, we are as yet unable to exclude this possibility that other *MADS-box* genes have roles in early fruit development (Table 2). Two Knotted-like homeobox (*KNOX*) genes, which can repress GA biosynthesis by the repression of *GA20ox* genes[28], *LeT6* and *LeT12*, were found down-regulation after fertilization and auxin/GA_3_ treatments(Table 2), which is in agreement with the higher expression of *GA20ox1*. Previous study in wild-type tomato and the *pat* mutant also acquired the same results[29]. It’s indicating that *LeT6* and *LeT12* might act as key negative regulators of fruit set.

A large number of genes involved in hormone biosynthesis and signaling were identified to be altered during fruit set (Fig 5A). Besides auxin and GA, ethylene was the most represented (Fig 5B). Previous study in *Arabidopsis* ethylene mutants showed that ethylene is involved in the modulation of the ovule senescence and the determination of the pistil fate during fruit set[30]. In our study, genes involved in ethylene biosynthesis (*ACOs*) and signaling (*ETR*, *EIN3*, *ERFs*) were down-regulated after fertilization and auxin/GA_3_ treatments (S6 Table). It is in accordance with the results in tomato performed by Vriezen [5] and in zucchini squash performed by Martínez [31]. Recently, Switzenberg et al.[32] reported that inhibition of ethylene perception ETR1 resulted in earlier fruit set and greater number of fruits. We also found that parthenocarpy occurred in *EIN3/EIL1 co-suppression* tomato lines (data not published). Taken together, ethylene is considered to be a key regulator in coordinating the process of fruit set.

### Regulation of pollination-dependent and parthenocarpic fruit set by auxin and GA

In the present study, we found that pollination and 2,4-D application can bring about the changes in the expression of the auxin signaling components, *Aux/ IAAs* and *ARFs*, while these genes were hardly regulated by the GA_3_ treatment e.g. *ARF9* (Table 3). Conversely, a GA synthesis gene, *GA20ox1* was strongly elevated by 2,4-D application. Moreover, GA signaling genes can be modulated by auxin (Table 3). These findings support the observation that auxin may act prior to GA during natural fruit initiation and auxin-induced fruit set is mediated in part by GA[2]. It has been reported that down-regulation of *IAA9* leads to parthenocarpy [12]. In accordance with this, *IAA9* transcript levels were decreased after pollination and GA_3_ treatment, but not in 2, 4-D treated ovaries (Table 3, S1 Table and S2 Table). Serrani et al. [13] also reported that increase in *IAA9* transcript level was exhibited in 2, 4-D treated ovaries. It is possible that 2, 4-D induced parthenocarpy is not mediated by *IAA9* down-regulation [13] and the parthenocarpic fruit set by *IAA9* silence is mediated by modified GA responses through auxin/GA crosstalk[8]. *ARF2*, identified to be involved in crosstalk between auxin and GA mediated by GID1, was suppressed after GA_3_ treatment (Table 3). Similarly, Richter et al [33] reported that GA treatment promotes ARF2: GFP abundance, but with a decrease in *ARF2* transcript level, which is explained by the hypothesis that ARF2 protein may repress its own transcription as part of a negative feedback regulatory loop. Therefore, we speculate that ARF2 plays a central role in fruit initiation, which is supported by our previous study that overexpression of *ARF2* results in an increase of fruit number and reduction of seed production (data not published). Interestingly, *ARF8B* was only modulated by GA_3_ application, with a decrease in its expression (Table 4, S1 Table). It’s in agreement with the fact that seedless fruits were obtained in *ARF8* loss of function mutants [9]. These findings indicate that GA-induced parthenocarpy may be mediated by *ARF2* and *ARF8* down-regulation.

A subset of genes associated with GA signaling was identified to be altered during ovary development. Six *GID1-like* genes, encoding putative GA receptors, were modulated during fruit set. While previous studies also showed *GID1* transcripts were regulated in response to pollination or GA application [6, 13]. GID2, an F-box protein that interacts directly with DELLA and mediates its degradation, is a positive regulator of GA signaling [34, 35]. Here, we reported that *GID2* transcript was repressed only by GA_3_ application (Table 4), and same result was recruited by Vriezen[5]. It seems that *GID2* expression is subject to GA feedback in order to sustain GA homeostasis. We also speculated that down-regulation of *GID2* may be involved in GA mediated parthenocarpy. DELLA, act as a GA repressor, is a key member in GA signaling. Reduction of *SlDELLA* mRNA levels induced parthenocarpy [36]. A member of DELLA gene family, *GAI*, was up-regulated after GA_3_ and 2, 4-D application in our dataset, which is in agreement with the results described by Serrani [13], suggesting feedback regulation. This result achieves equal outlook that DELLA proteins mediate repression of *DELLA* transcription[37]. In addition, other GRAS family members without DELLA domain draw attention regarding their significant down-regulation (Table 3). However, it is noted that the roles of them on GA response and fruit development are largely unknown.

Pagnussat et al.[38] reported that the interaction of BLH1 and KNAT3 was considered to compose functional complexes regulating normal development and cell specification in the *Arabidopsis* embryo sac. In the present study, auxin application resulted in a dramatic decline in the expression of *KNAT3*. And *BLH1* displayed down-regulation during both auxin-and GA-mediated fruit set (Table 4). Moreover, parthenocarpy was induced in *BLH1* loss of function mutants [39]. Hence, auxin/GA induced parthenocarpy might be mediated by BLH1-KNAT3 interaction. In addition, *AGL66* transcript was found to be repressed (Table 4), which was also occurred in parthenocarpy lines with *PIN4* silence, indicating its potential role in the formation of seedless fruits[7]. We also found that *AGO10* and *AGO5* were significantly down-regulated (Table 4). AGO10, as a critical regulator of SAM maintenance, functions by interacting miR165/166. It has been reported that miR166 levels are increased in *ago10* loss of function mutants [40]. We hypothesis that down-regulation of *AGO10* causes miR166 accumulation in early developing ovaries and subsequent regulation of fruit growth driven by target genes. Furthermore, it has been revealed that overexpression of *miR166* by a fruit specific promoter p2A11 results in a remarkable shift in tomato carpel development, that is production of secondary fruits at the apex and seedless fruits (data not published). Therefore, the results indicate the major roles of these genes in parthenocarpic fruit set.

## Conclusions

**Fig 7:**
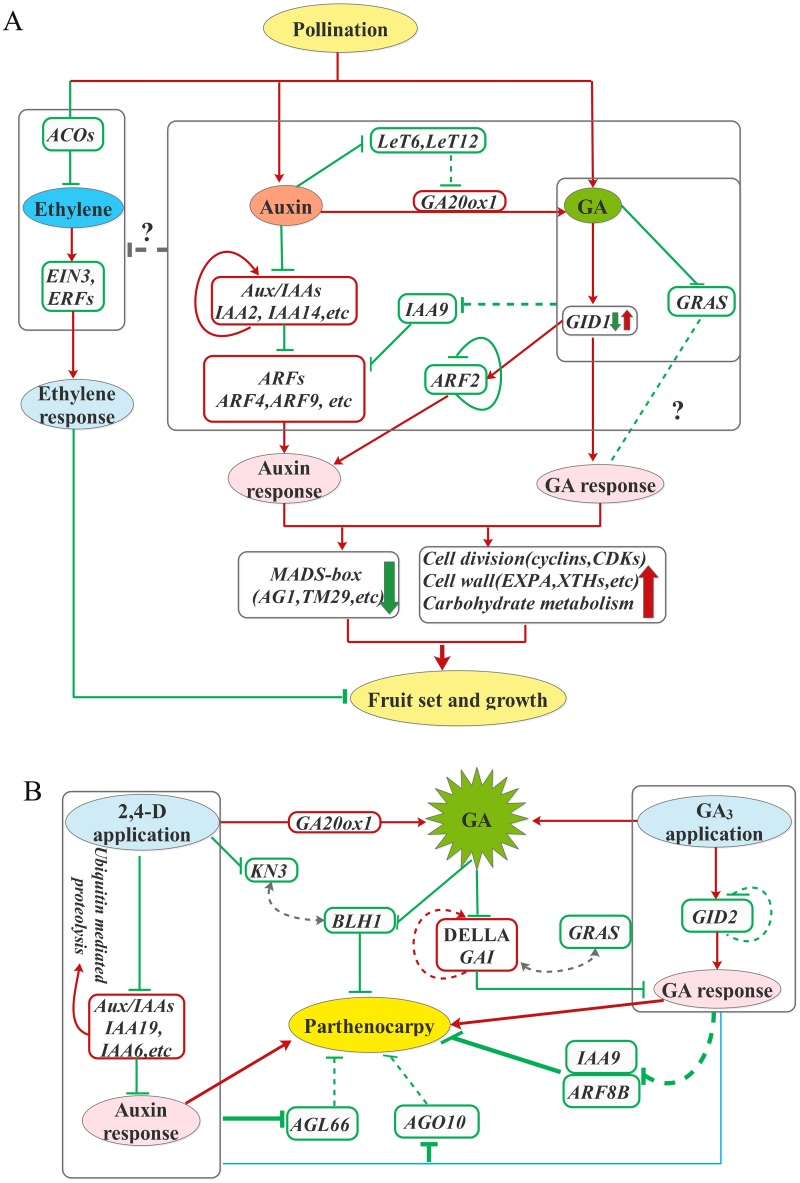
Model for regulatory events underlying fruit set mainly mediated by auxin and gibberellin. (A) After pollination, the increase of auxin and GA biosynthesis and response will trigger fruit initiation. And the elevated auxin and GA would inhibit ethylene biosynthesis and response which negatively regulate fruit set. Auxin stimulates GA biosynthesis gene *GA20ox1* via down-regulating *KNOX* (*LeT6*, *LeT12*) in partially, subsequently influences GA response by regulating *GID1* and *GRAS*. Some Aux/IAAs may be degraded by the 26S proteasome and in turn, the transcripts are increased via feedback regulation to fine-tune auxin response. Some ARFs (*ARF4*, *ARF9*) are up-regulated which could promote auxin signaling cascades. In addition, auxin response may be mediated by GA via down-regulation of *IAA9* and *ARF2* during fruit set. Accordingly, events that the activation of carbohydrate metabolism, cell division and expansion as well as the down-regulation of *MADS-box* occur to promote fruit set process. (B) After 2, 4-D application, *KNAT3* displayed down-regulation, which would interact with BLH1, thus negatively regulating parthenocarpy. In the present of GA, the transcripts of *DELLA* and *GID2* are regulated via feedback to fine-tune GA response. GA application decreased the transcripts of *IAA9* and *ARF8B*, and then led to parthenocarpy. Moreover, other factors such as *GRAS*, *AGL66* and *AGO10*, which are down-regulated after hormone application, also seem to have a regulatory role in parthenocarpy. The red and green frame shows up- and down-regulation, respectively. The red line arrow and the green line behind represent positive and negative regulation, respectively. The regular arc line represents feedback regulation. Dashed line shows suggested interactions.

In summary, regulatory events underlying pollination-induced and parthenocarpic fruit set are revealed in our work. The model presented in Fig 7 highlights the roles of auxin and GA on fruit set and the crosstalk between the two hormones. We proposed that the effect of auxin on pollinated dependent and parthenocarpic fruit set is mediated by GAs to some extent. Auxin can elevate GA biosynthesis gene *GA20ox1*, which may be partially attributed to the down-regulation of *KNOX* genes that act as negative regulators on *GA20ox1*, thus control GA responses. Alternatively, the crosstalk between auxin and GA involves in *ARF2* and *IAA9* down-regulation. In contrast to pollination, a much stronger activation of *Aux/IAAs* and feedback of *GID2* and *DELLA* in their transcripts displayed after 2, 4-D and GA_3_ application respectively, suggesting their roles in fine-tuning hormone response and regulating parthenocarpic fruit set. It is likely that whilst auxin and GA-mediated parthenocarpy act in partially distinct cascades, some pathways are common to pollination and hormones induced fruit set. Of particular note was the concerted activation of carbohydrates metabolism, cell division and expansion as well as the down-regulation of transcription factors especially in MADS-box after pollination or hormone application, suggesting that they may functioned as key components in regulating fruit initiation and ovary development. In addition, ethylene is considered to be a key regulator in coordinating the process of fruit set. In the further work, metabolomics tools would be employed to analysis the metabolic changes during fruit set, which is coupled with our transcriptome data in order to elucidate the mechanism of seed formation in pollinated ovaries and differences in morphology between auxin and GA triggered seedless fruits.

## Materials and Methods

### Plant materials and growth conditions

Tomato plants (*Solanum lycopersicum* L. cv micro-tom) were used in the experiments. Seeds were germinated in 1/2 MS medium, and then transplanted to 6 L pots containing a mixture of peat: sludge: perlite(6:1:1). Plants (four per pot) were cultured in a greenhouse under standard conditions (25°C/20°C day/night temperature,80% humidity and natural light was supplemented with lamps to get a 14 h photoperiod) and supplied with 500 mL of Hoagland solution every 7 days.

Flower emasculation was carried out 2 days before anthesis to avoid self-pollination. Artificial pollination was performed on the day just equivalent to anthesis. The ovaries were collected as samples for RNA-seq at the 2 days ahead of anthesis (2DAA, control) and 4 days after artificial pollination (4DPAP). Besides, the ovaries at 0, 2, 6, 8 and 10 days after artificial pollination were collected (0 DPA, 2 DPAP~10 DPAP).

### Application of auxin and gibberellin

According to Serrani et al. [11], unpollinated ovaries responded to different auxins (IAA, NAA and 2,4-D), 2,4-D being the most efficient. Serrani et al.[3] also revealed that unpollinated ovaries developed parthenocarpically in response to GA_3_ > GA_1_ = GA_4_ > GA_20_. Therefore, 2,4-D and GA_3_ (Sigma Aldrich, USA) were used in this study. Briefly, 2,4-D and GA_3_ were dissolved in ethanol as storage solution, then diluted with distilled water including 5% ethanol and 0.1% Tween 80 when worked to the concentration of 2,4-D and GA_3_ at 0.05mM and 0.3mM, respectively. The stigmas were treated with 2,4-D and GA_3_ in 10μL solution on the day just equivalent to anthesis. After 4 days, the uniform ovaries were collected and named as 4DPAT and 4DPGT which represented ovaries obtained from 4 days after auxin and GA_3_ treatment, respectively. Meanwhile, the untreated ovaries (6DPE) were collected as another control. For samples collection, three biological replicates were performed (40 ovaries on controls and 15 ovaries on the other treatments approximately in each replicate). All collected samples were frozen in liquid N_2_ and stored at -80°C until RNA-Seq experiment.

### RNA-Seq analysis

Total RNA from ovary samples including 2DAA, 6DPE, 4DPAP, 4DPAT and 4DPGT was extracted using Trizol reagent (Invitrogen, USA) according to manufacturer’s instruction. Poly A RNA purification, cDNA synthesis, tag preparation and RNA-Seq were performed by technicians in line with specified experimental process at Beijing Genome Institute (BGI) (Shenzhen, China). Briefly, mRNA are enriched through beads of Oligo (dT) and transferred into double-stranded cDNA via reverse transcription. cDNA is digested with *Nla*III which cut off CATG sites and Illumina adaptor 1 is ligated to the 5’ end of fragments. Then, *Mme*l is used to digest at 17 bp downstream of CATG site and Illumina adaptor 2 is ligated at 3' end. Finally, 95 bp fragments produced by PCR amplification are purified through 6% TBE PAGE and sequenced using Illumina HiSeq 2000 system.

### Sequence assembly and gene annotation

The Raw sequences were filtered by removing 3' adaptor fragments as well as low quality sequences (tags with unkown sequence“N”) and several types of impurities which are too long or too short and with a copy number of 1. After that, raw reads were transformed into clean tags. To identify the gene expression signatures from the four types of tomato libraries, the SGN unigene database (ftp://ftp.sgn.cornell.edu/unigene_builds/combined_species_assemblies/tomato_species/curr/tomato_species_unigenes.seq.gz) was used as a reference database. All clean tags were aligned to the reference sequences using SOAP[41], only allowing 1 bp mismatch. Clean tags with multiple matches were excluded. The number of unambiguous clean tags for each gene was calculated and then normalized to TPM (number of transcripts per million clean tags) [42, 43].

### Screening of differentially expressed genes

R package DEGseq was applied to identify differentially expressed genes with random sampling model [44]. A P-value could denote its expression difference between two libraries, and false discovery rates (FDRs) were used to determine the threshold of P value. We set "FDR≤0.001 and the absolute value of log2Ratio≥1" as the threshold to judge the significance of gene expression difference according to described method [45]. Cluster analysis of differentially expressed genes was performed using "cluster" [46] and "Java Treeview" [47] software. The value of log2 ratio was used for the hierarchical clustering analysis. Gene ontology (GO) terms of differentially expressed genes were identified by the software Blast2GO (version 2.3.4) [48] using the default parameters. The hypergeometric test was applied to perform GO enrichment analysis of functional significance when all differentially expressed genes were mapped to terms in GO database. Blast2GO was also employed to identify significantly altered pathways during fruit set. Pathways with Q value≤0.05 are considered significantly enriched in DEGs.

### Sequence deposition

The raw transcriptome reads reported here have been deposited in the NCBI Short Read Archive under accession Nos. SRX850785 (2DAA), SRX850788 (4DPAP), SRX850793 (4DPAT), SRX850794 (4DPGT) and SRX850795 (6DPE).

### Validation of RNA-seq data by quantitative real-time PCR

Total RNA was extracted using TRIzol reagent (Invitrogen,USA) from different ovary samples. cDNA was synthesized from 2 μg of total DNA-free RNA using RevertAid First Strand cDNA Synthesis Kit (Fermentas, USA) according to the manufacturer’s instruction and diluted five folds as the template. Gene-specific primers for selected genes were designed by online software (https://www.genscript.com/ssl-bin/app/primer) (S7 Table). And melt curve analysis was used to confirm the specificity. Quantitative real-time PCR (qRT-PCR) were carried out using SsoFast EvaGreen Supermix (Bio-Rad, USA) on an Bio-Rad CFX96 real-time PCR detection system by the three-step method, which was incubated at 95°C for 5 min, then followed by 40 cycles of 95°C for 5 s, 60°C for 5 s and 72°C for 5 s. For qPCR experiments, three biological replicates were performed. Relative expression levels were calculated based on the 2^-ΔΔCt^ method using actin as reference gene.

## Supporting Information

S1 Fig Saturation evaluation and distribution analysis of the RNA-Seq tags in the five libraries. (TIF)

S2 Fig Functional categorization of DEGs during fruit set based on biological process of Gene Ontology. (TIF)

S3 Fig The expressions of selected genes in ovaries after artificial pollination. (TIF)

S4 Fig Cluster analysis of DEGs related to hormone during fruit set. (TIF)

S5 Fig Cluster analysis of DEGs related to transcription factors. (TIF)

S1 Table The DEGs in ovaries during fruit set with respect to 2DAA in tomato. (XLSX)

S2 Table The DEGs in ovaries during fruit set with respect to 6DPE in tomato. (XLSX)

S3 Table The common and distinct DEGs during the three types of fruit set in tomato. (XLSX)

S4 Table DEGs involved in photosynthesis and carbohydrate metabolism during fruit set. (XLSX)

S5 Table DEGs involved in cell division and cell wall metabolism during fruit set. (XLSX)

S6 Table DEGs involved in hormone biosynthesis and signaling during fruit set. (XLSX)

S7 Table Primers used for qRT-PCR and the accession numbers of the relevant genes. (XLSX)
