# Supplementary figures and images for: Transcriptome Profiling Reveals the Regulatory Mechanism Underlying Pollination Dependent and Parthenocarpic Fruit Set Mainly Mediated by Auxin and Gibberellin
Source: PLoS One. 2015 Apr 24;10(4):e0125355. doi: 10.1371/journal.pone.0125355 (PMC4409352; doi:10.1371/journal.pone.0125355)

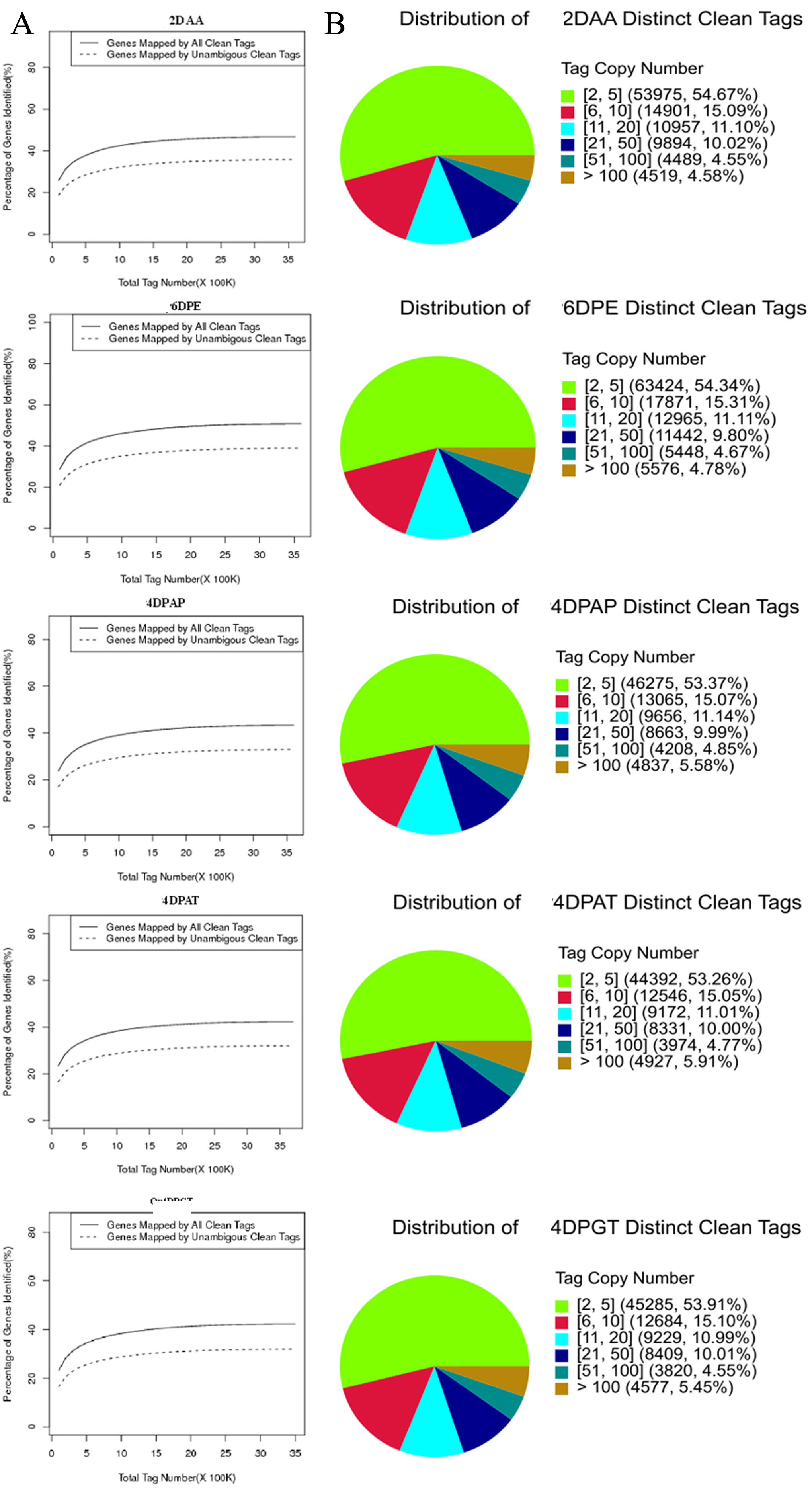

Supplement: S1 Fig — (TIF) [file pone.0125355.s001.tif]

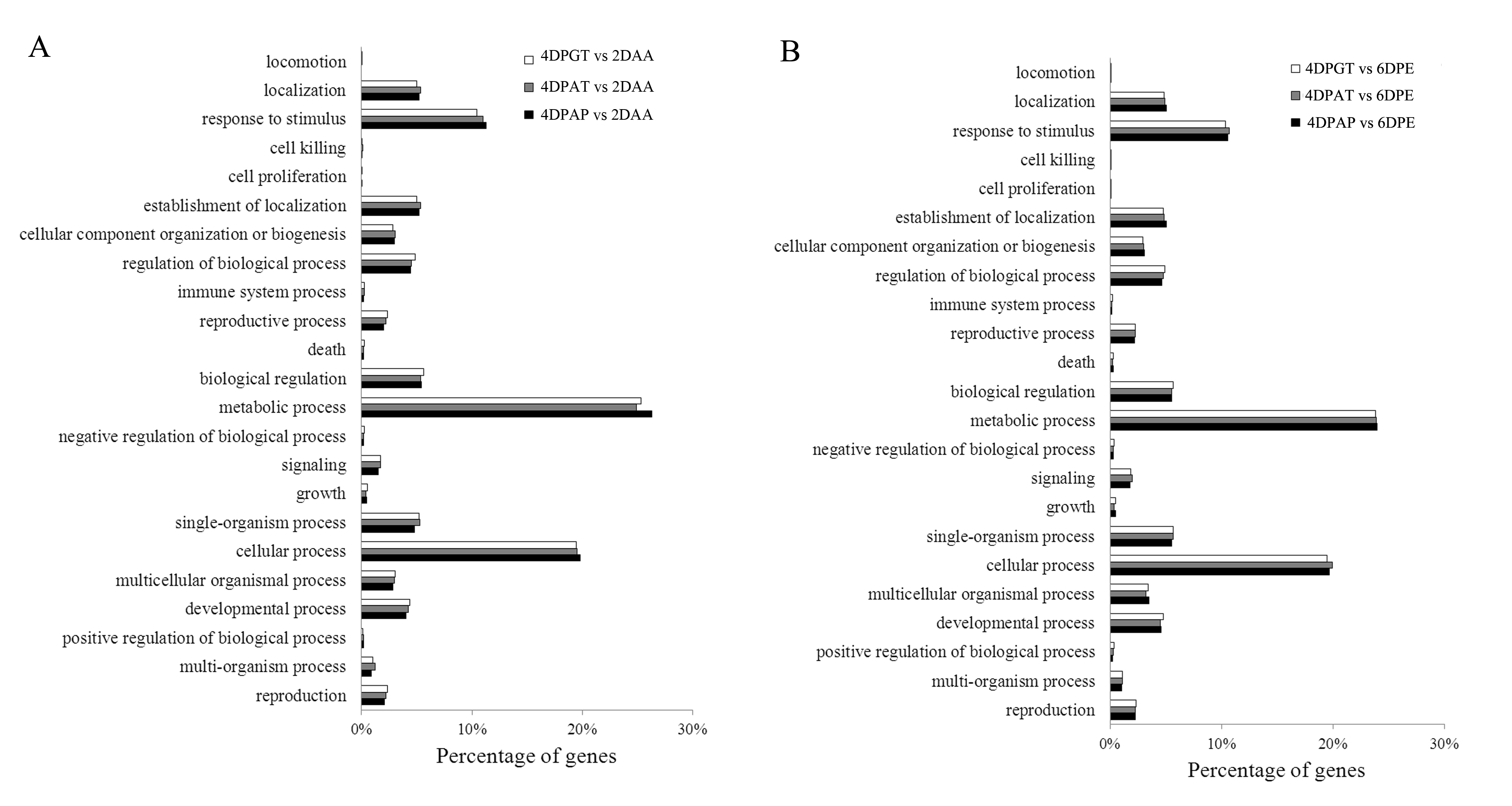

Supplement: S2 Fig — (TIF) [file pone.0125355.s002.tif]

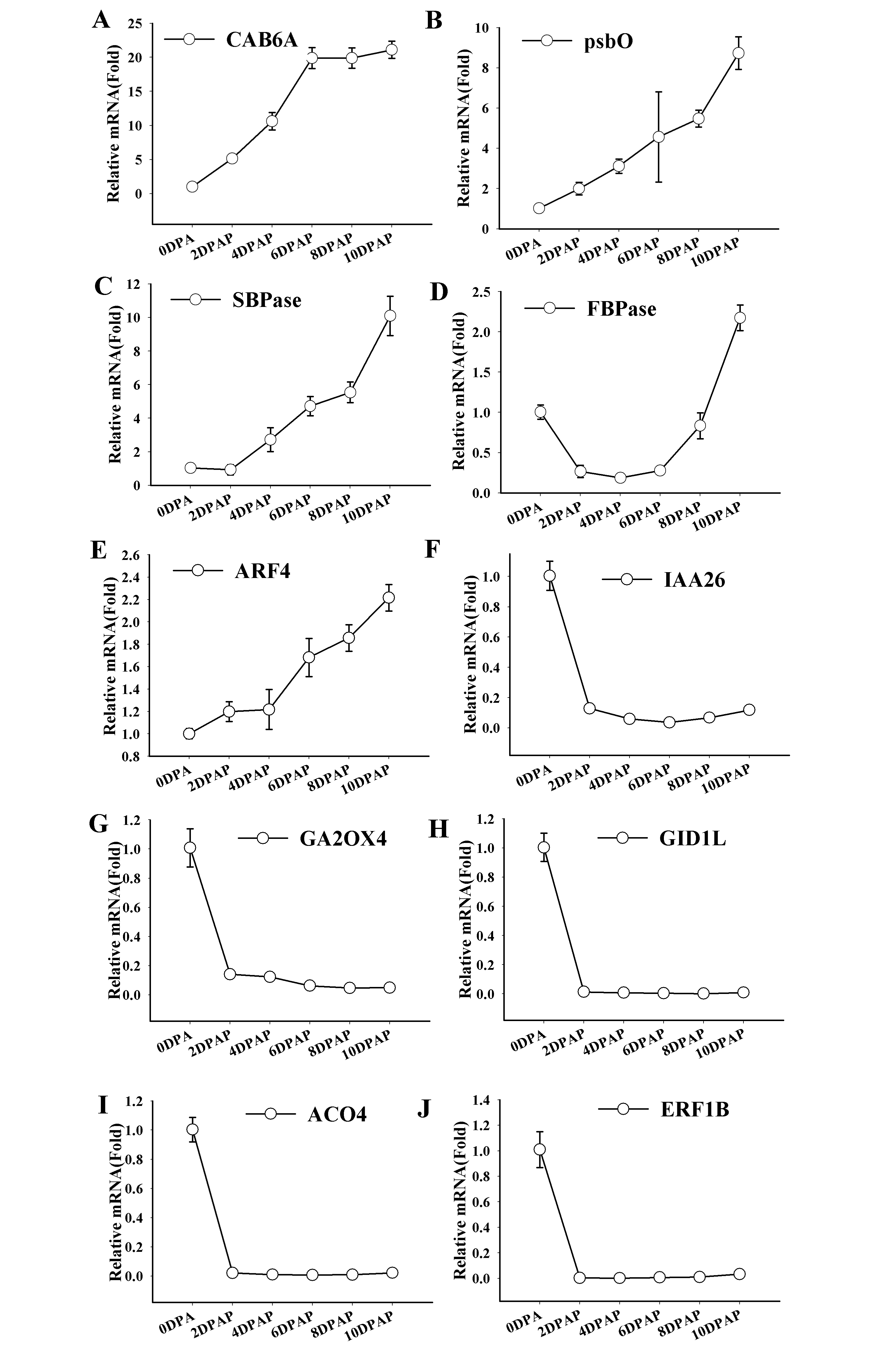

Supplement: S3 Fig — (TIF) [file pone.0125355.s003.tif]

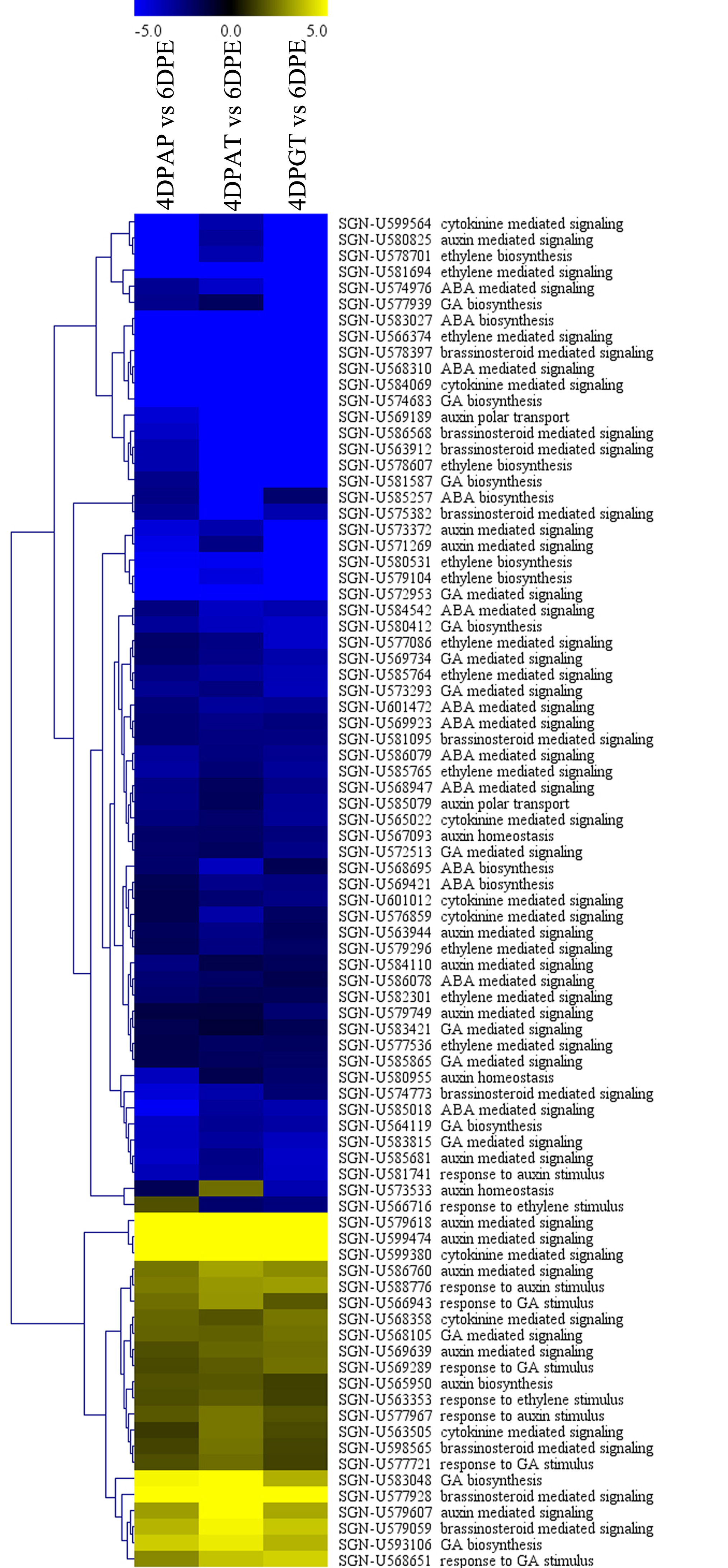

Supplement: S4 Fig — (TIF) [file pone.0125355.s004.tif]

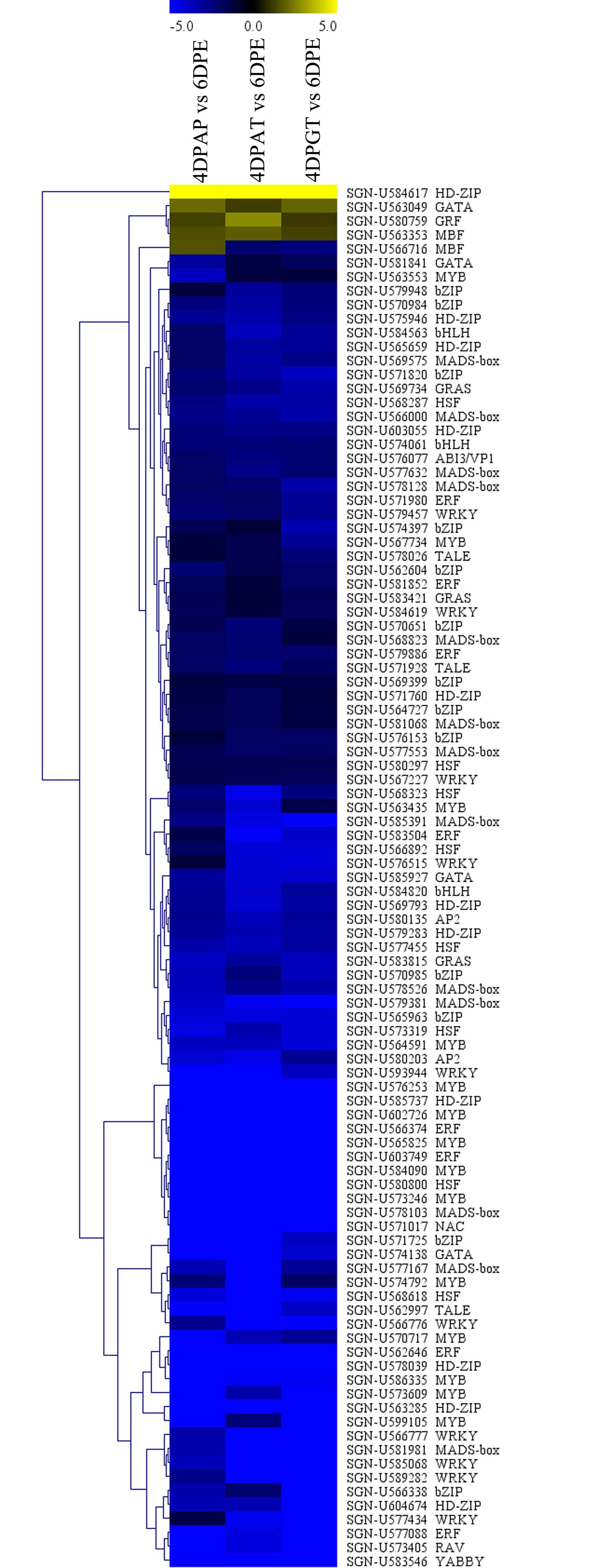

Supplement: S5 Fig — (TIF) [file pone.0125355.s005.tif]
